# Malicious and Benign Webpages Dataset

**DOI:** 10.1016/j.dib.2020.106304

**Published:** 2020-09-12

**Authors:** A.K. Singh

**Affiliations:** Advanced Data Analytics & Parallel Technologies Lab (ADAPT Lab), Department of Computer Science & Information Systems, BITS Pilani, Pilani Campus, India

**Keywords:** Web security, Malicious webpages, Machine learning, Deep learning, Malicious JavaScript

## Abstract

Web Security is a challenging task amidst ever rising threats on the Internet. With billions of websites active on Internet, and hackers evolving newer techniques to trap web users, machine learning offers promising techniques to detect malicious websites. The dataset described in this manuscript is meant for such machine learning based analysis of malicious and benign webpages. The data has been collected from Internet using a specialized focused web crawler named MalCrawler [1]. The dataset comprises of various extracted attributes, and also raw webpage content including JavaScript code. It supports both supervised and unsupervised learning. For supervised learning, class labels for malicious and benign webpages have been added to the dataset using the Google Safe Browsing API.^1^ The most relevant attributes within the scope have already been extracted and included in this dataset. However, the raw web content, including JavaScript code included in this dataset supports further attribute extraction, if so desired. Also, this raw content and code can be used as unstructured data input for text-based analytics. This dataset consists of data from approximately 1.5 million webpages, which makes it suitable for deep learning algorithms. This article also provides code snippets used for data extraction and its analysis.

## Specifications Table

**Table:** 

Subject	Artificial Intelligence
Specific subject area	Machine Learning using Web Content
Type of data	Dataset Tables Figures Graphs Python Code
How data were acquired	The data were collected from the Internet by scraping webpages using a customized focused web crawler named MalCrawler [1]. Thereafter, the raw data collected was processed using customized Python code to extract relevant features.
Data format	Raw (Unstructured web content and JavaScript) Analyzed Filtered
Parameters for data collection	Web content was pruned down to reduce size by removing less relevant content, viz., meta data, stop words, style data, HTML tags, etc. Obfuscated JavaScript code was de-obfuscated using a browser emulator.
Description of data collection	The raw data comprises of webpages. This data was collected from the Internet by scraping websites using MalCrawler [1]. MalCrawler is a focused crawler designed to seek more malicious webpages compared to a random web crawl. Scraped data was further processed using customized Python code to extract attributes. Class labels for malicious and benign webpages were added using the Google Safe Browsing API [2].
Data source location	Data was gathered from Web between November 2019 and March 2020, with random web crawls carried out to ensure adequate global coverage.
Data accessibility	Data hosted in public repository. Repository name: Mendeley Data Data identification number: 10.17632/gdx3pkwp47.2 Direct URL to data: http://dx.doi.org/10.17632/gdx3pkwp47.2

## Value of the Data

• Useful for building machine learning models for carrying out varied analysis on webpages. Both supervised and unsupervised learning models can be developed. It is pertinent to note that presently no such comprehensive dataset exists in public domain to facilitate research work in this field.
• Will benefit all researchers who are pursuing research in the field of Web Security. Further, this data can be used by Cyber Security firms or Anti-Virus companies to model their security products.
• Contains sufficient attributes for further insight and future work. Notwithstanding, this data also includes processed raw web content, including JavaScript code, which can be used for extraction of new attributes, if so required, to aid future research.
• It has value, not only to Internet Security research community or Cyber Security firms, but can also be used for policy development by Cyber Law Enforcement agencies.

## 1 Data Description

The dataset was designed and prepared with the aim of classification of webpages as Malicious or Benign. However, this dataset contains sufficient information that can be used for any machine learning task related to webpage analysis. The attributes of this dataset are listed below in Table 1.

**Table 1:** Attributes of dataset.

#	Attribute name	Data type	Attribute description
1.	url	String	URL of the Webpage.
2.	ip_add	String	IP Address of the webpage.
3.	geo_loc	Categorical String {Variable Bucket Size}	Name of the country based on IP Address location.
4.	url_len	Numerical {int16}	Length of URL- count of characters in a URL.
5.	js_len	Numerical {float64}	Length of JavaScript code (in KB) in the webpage.
6.	js_obf_len	Numerical {float64}	Length of Obfuscated JavaScript (in KB) in the webpage.
7.	tld	Categorical String {Variable Bucket Size}	Top Level Domain of the webpage.
8.	who_is	Categorical String {Value- incomplete/complete}	Gives out whether the WHO IS information of the registered domain is complete or incomplete.
9.	https	Categorical String {Value- yes/no}	Gives out whether the website uses https or http protocol.
10.	content	Text	Raw Web Content of the Webpage. Includes filtered and processed text and JavaScript code.
11.	label	Categorical String {Value- good/bad}	Classification label categorizing the webpage class as Malicious (bad) or Benign (good).

**Fig. 1:**
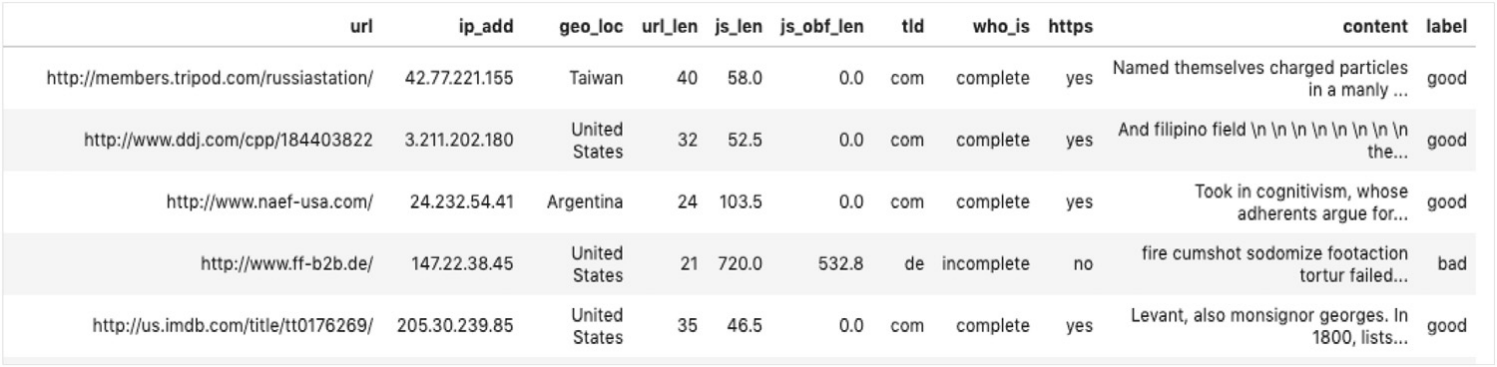
Snapshot of the dataset.

The dataset comprises of 1.564 million webpages having 11 attributes. These attributes were selected based on their performance in predicting malicious and benign webpages in previous researches [5]. A snapshot of the dataset is shown below in Fig. 1.

**Fig. 2:**
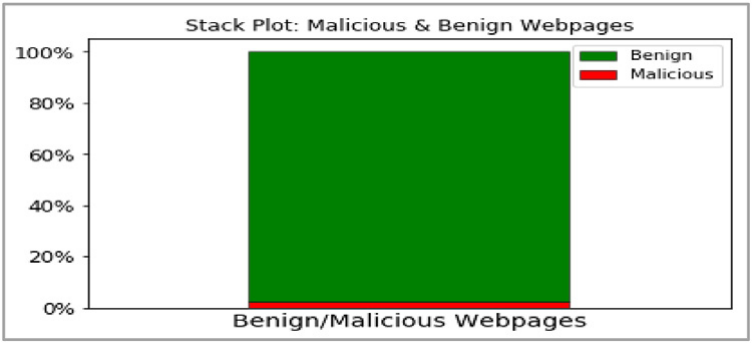
Class label distribution- Malicious & Benign.

The last attribute in Table 1 is Class Label, which can be used for training the machine learning algorithm. The two classes correspond to Malicious and Benign webpages. As the Internet has more Benign pages than Malicious^2^ webpages, a similar disproportion also reflects in our Dataset. As seen in the graphical representation of Class Labels in Fig. 2, a majority of the webpages are benign. Thus, users of this dataset should appropriately factor this skew in class distribution while training machine learning models.

**Fig. 3:**
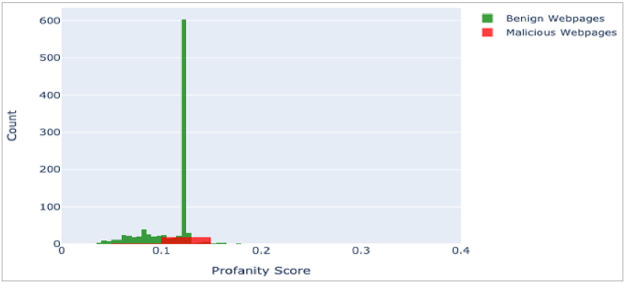
URL plot (vectorized using profanity score).

**Fig. 4:**
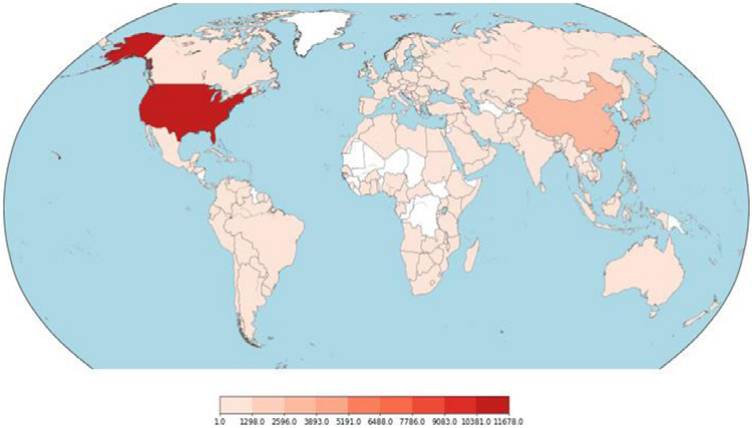
Geographic distribution of IP addresses - Malicious.

**Fig. 5:**
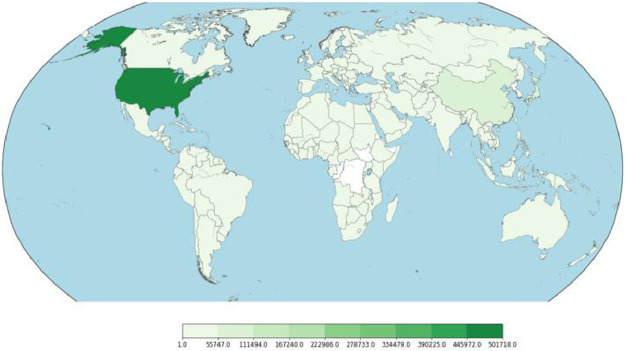
Geographic distribution of IP addresses - Benign.

First attribute of the dataset represents URL of the webpages. Visualization of ‘url’ attribute, after vectorizing it (using Profanity Score^3^), is depicted in Fig. 3. The second attribute ‘ip_add’ gives the IP Address of the Webserver hosting the webpage. Third attribute ‘geo_loc’ gives the country to which the IP Address belongs. The IP Address distribution is plotted country wise in Fig. 4 and Fig. 5 for Malicious and Benign webpages, respectively. As can be inferred from the maps in Fig. 4 and Fig. 5, the dataset represents webpages from servers across the globe.

**Fig 6:**
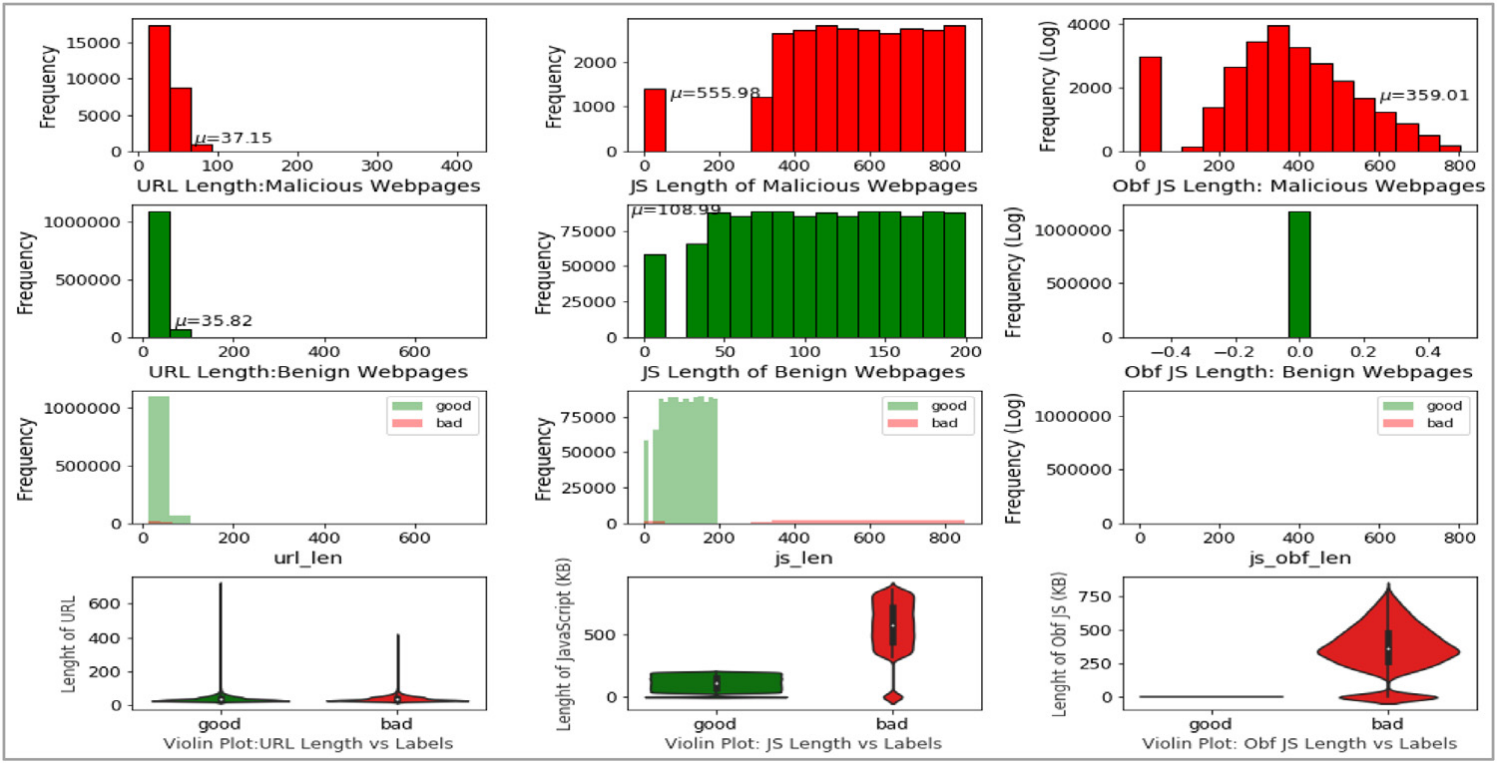
Univariate plots: URL length, JavaScript length and obfuscated JavaScript length.

The fourth, fifth and sixth attribute of the dataset are ‘url_len’, js_len’ and ‘js_obf_len’ respectivley. All three are numerical attributes and their univariate plots are shown below in Fig. 6.

**Fig. 7:**
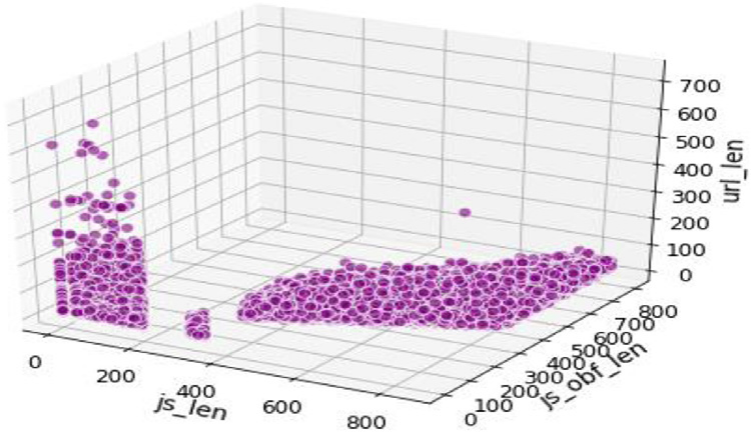
Trivariate 3D plot.

**Fig. 8:**
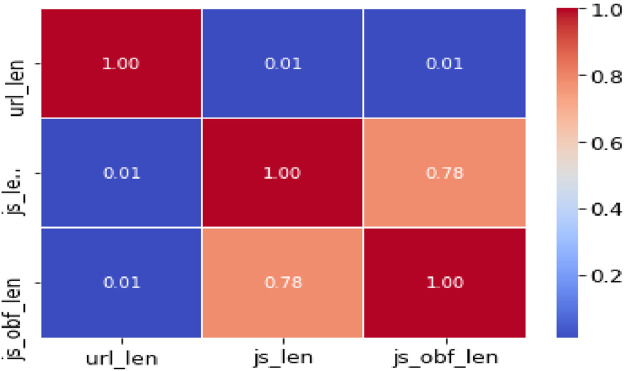
Trivariate correlation matrix.

**Fig. 9:**
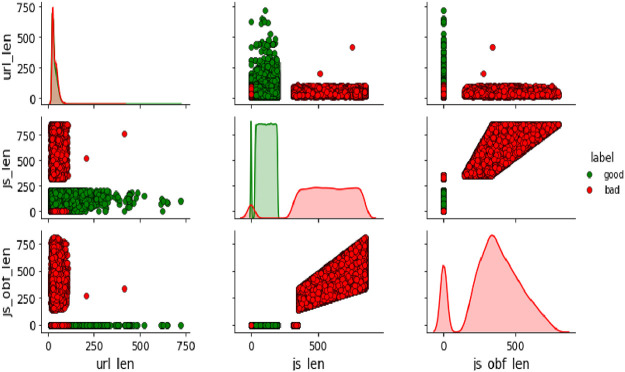
Trivariate pairwise plot.

**Fig. 10:**
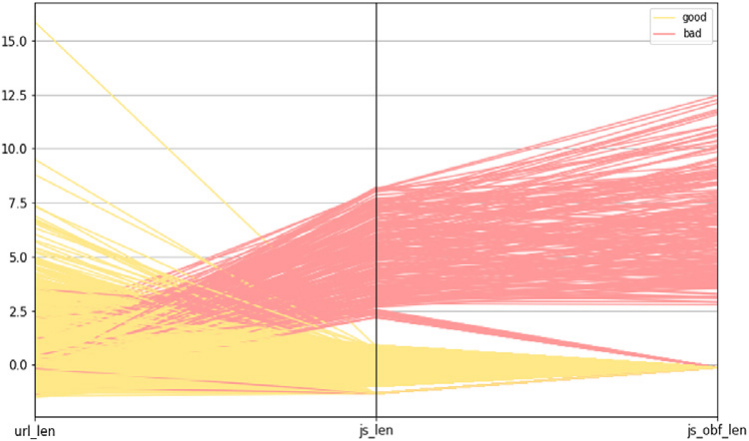
Trivariate parallel coordinates plot.

The trivariate distributions of these three numerical attributes are shown in Fig. 7, Fig. 8, Fig. 9, Fig. 10. Fig. 7 gives the 3D plot, Fig. 8 shows correlation score^4^ amongst these three numerical attributes, Fig. 9 plots these three attributes against each other pairwise, and Fig.  10 plots all three together as parallel coordinates.

**Fig. 11:**
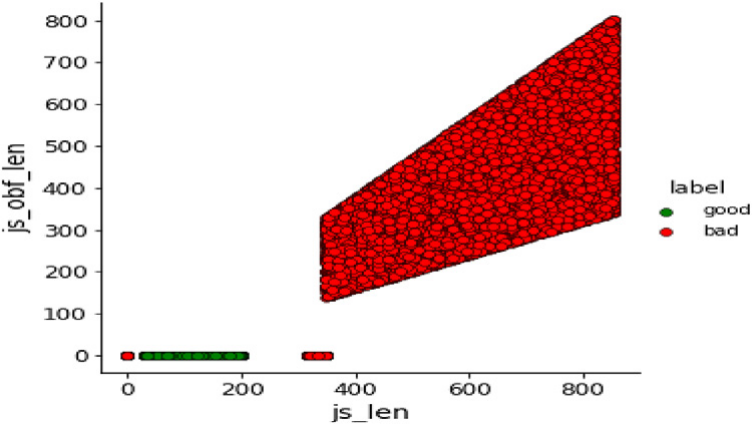
Bivariate pairwise plot.

**Fig. 12:**
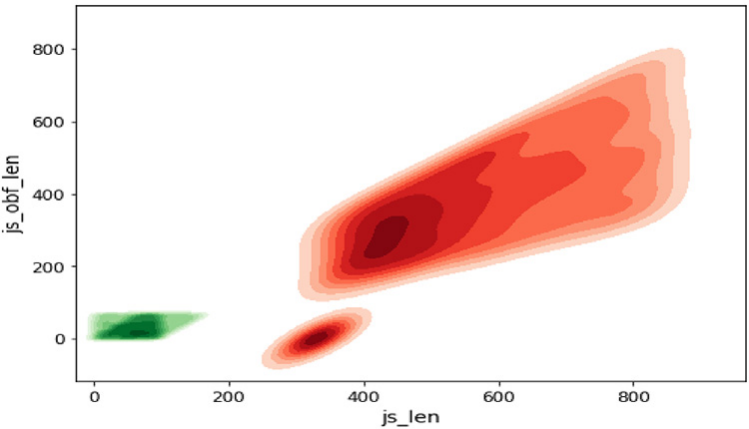
Bivariate density plot.

As attributes ‘js_len’ and ‘js_obf_len’ have exhibited high correlation in matrix of Fig. 8, their bivariate distributions are plotted in Figs. 11 and 12 to highlight their relationship.

**Fig. 13:**
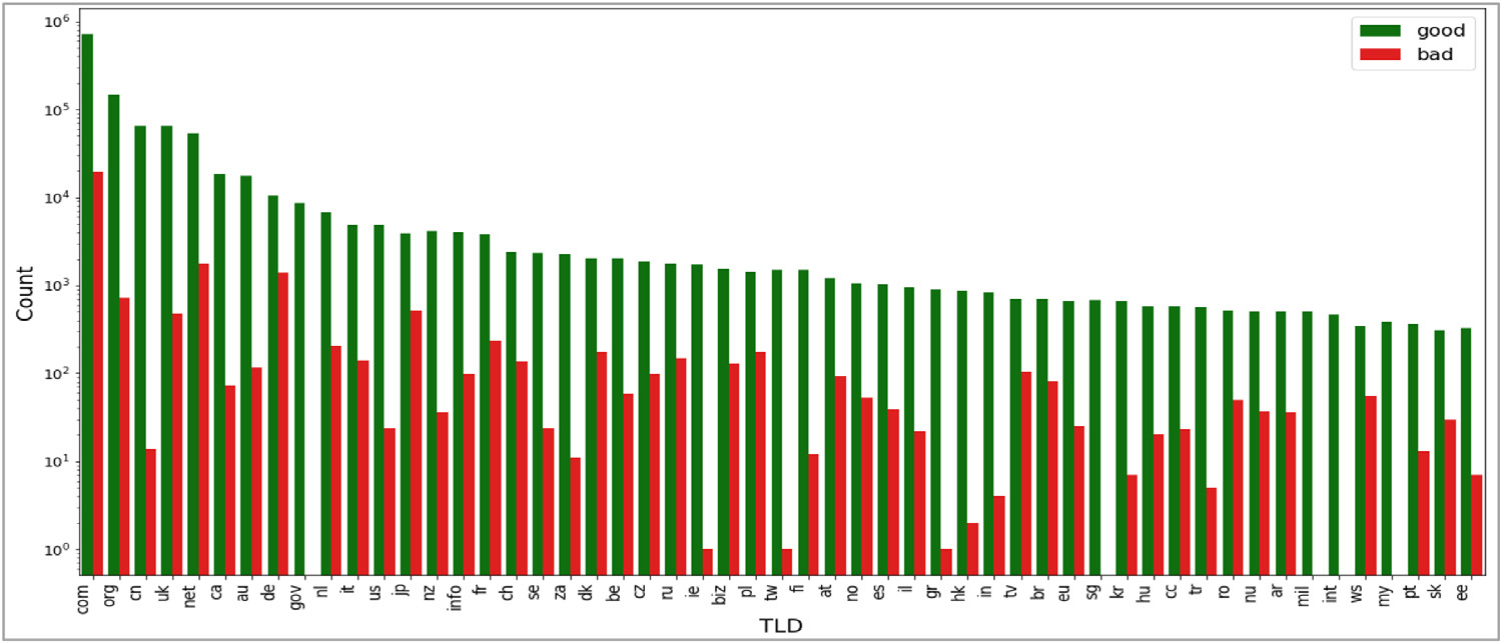
Plot of top level domain (‘tld’) attribute.

The seventh attribute is ‘tld’ that gives the Top Level Domain Name of the webpage. This attribute is plotted in Fig. 13. As depicted by the graph, this dataset contains webpages from numerous domains.

**Fig. 14:**
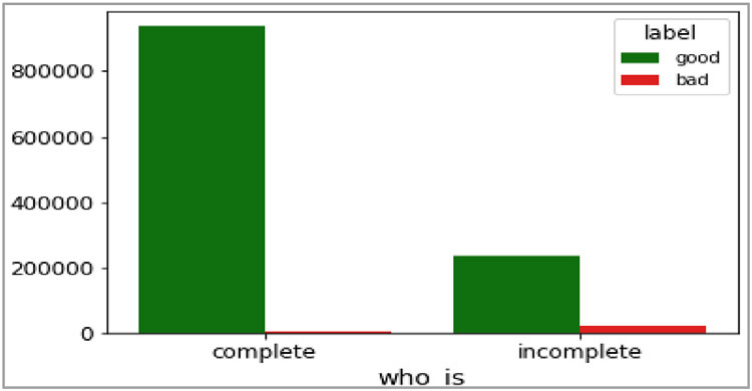
Plot of who is registration (‘who_is’) attribute.

**Fig. 15:**
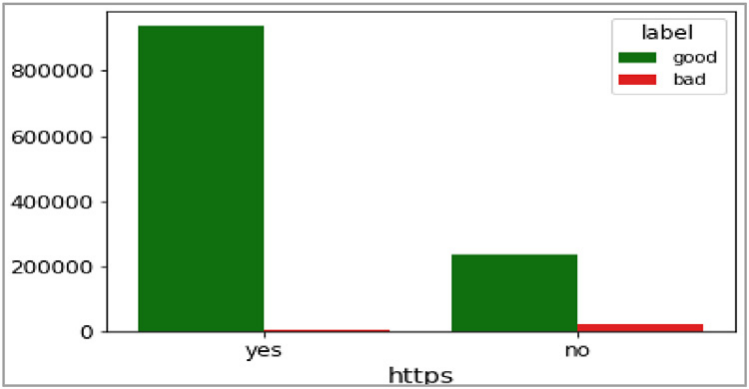
Plot of HTTPS (‘https’) attribute.

The eighth and ninth attributes of dataset are ‘who_is’ and ‘https’ respectively. Both are categorical attributes. The ‘who_is’ attribute gives completeness of domain registration records of websites, which are held with domain registrars. The ‘https’ attribute tells us whether HTTP secure protocol is used by the webserver or not for delivering the webpage. These two attributes are visualized in Figs. 14 and 15 below.

**Fig. 16:**
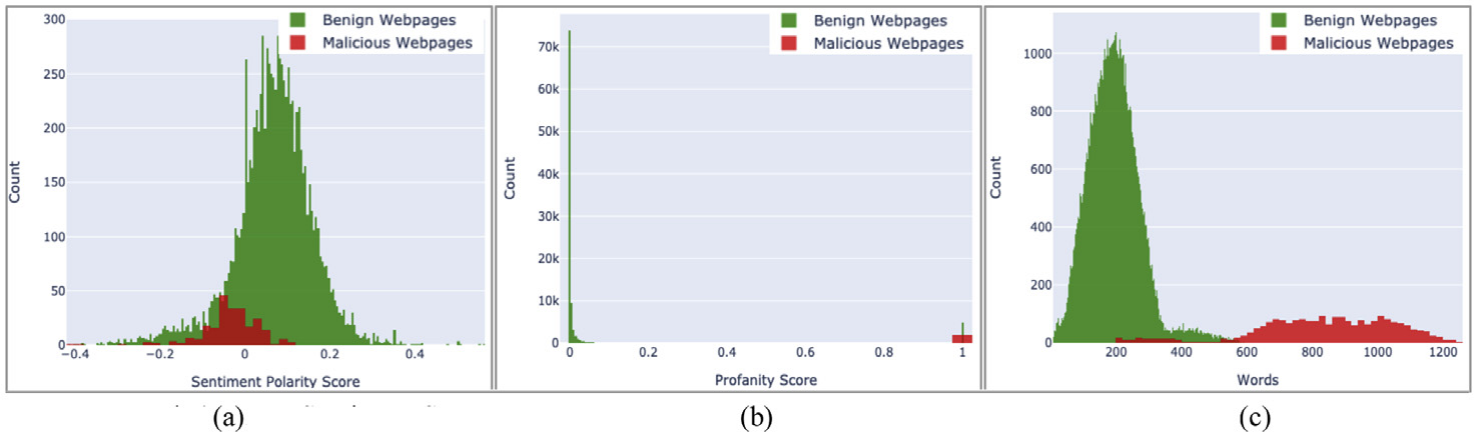
(a) Web content: sentiment score. (b) Web content: profanity score. (c) Web content: word count.

The tenth attribute of the dataset is ‘content’. This attribute contains raw web content, including JavaScript code, which has been filtered and cleaned to reduce size. The objective of providing this attribute in the dataset is to enable further attribute extraction from this dataset, if so desired in future research. Further, certain machine learning techniques, like Deep Learning, can use this unstructured web content directly for experiments. Fig. 16(a), (b) and (c) below show the vectorized plot of this raw content.

**Fig. 17:**
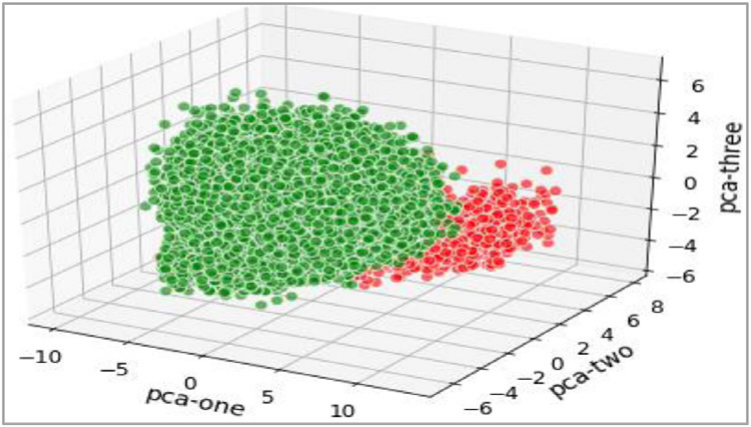
3D plot of complete dataset.

**Fig. 18:**
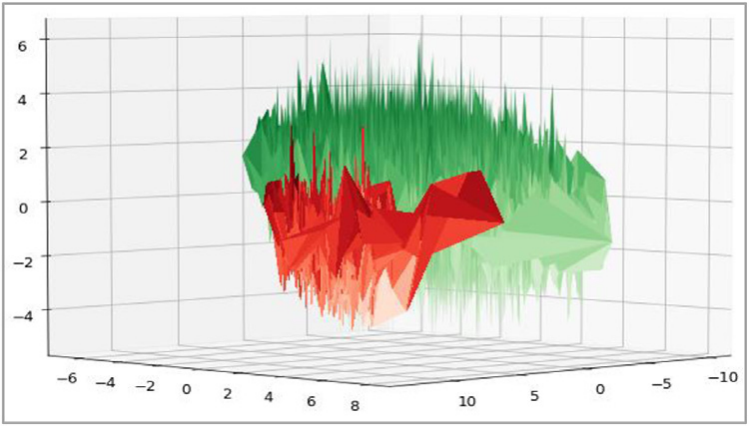
Tri surface plot of complete dataset.

All attributes discussed above, reduced to three dimensions using Principal Component Analysis (PCA) are plotted below. The 3D scatter plot is given below in Fig. 17, while the Tri Surface plot is given in Fig. 18. These plots show that the dataset is non-convex; however, it can be segregated into classes. Thus, data scientists can apply various machine learning techniques to this dataset.

The objective of showing the above visualizations of dataset and its attributes is to enable readers of this article to understand the dataset better, and accordingly utilize it for their research. The detailed visualization, with more insight and analysis, along with the Python code that has been used to generate it, is available alongside the dataset hosted on Mendeley repository [3]. Also, the visualization output is hosted publicly on Kaggle for live experimentation [4].

## 2 Experimental Design, Materials, and Methods

**Fig. 19:**
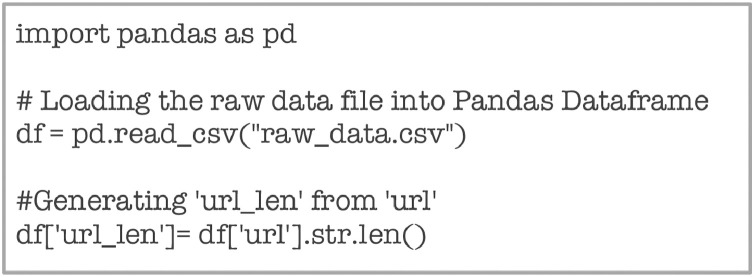
Code snippet for extracting ‘url_len.

The dataset was collected by scraping websites across the globe on the Internet. MalCrawler [1], which is a special purpose focused crawler, was used for this task. MalCrawler [1] is a preferred crawler for this task as it seeks more malicious websites than a random crawl by any other generic web crawler. Further, it is a uniquely designed crawler that does not get entangled in deep crawls or in dynamic websites. The data collected from crawl was then processed to extract the attributes, which have been described in the previous section. The basic information captured during the crawl included IP address, URL, and web content. Other attributes were thereafter extracted using customized Python Code. The choice of attributes extracted for this dataset was based on its relevance in malicious webpage classification, as brought out by Singh et al. in their paper [5]. The attribute ‘url_len’ was computed from ‘url’ using the Python code given in Fig. 19.

**Fig. 20:**
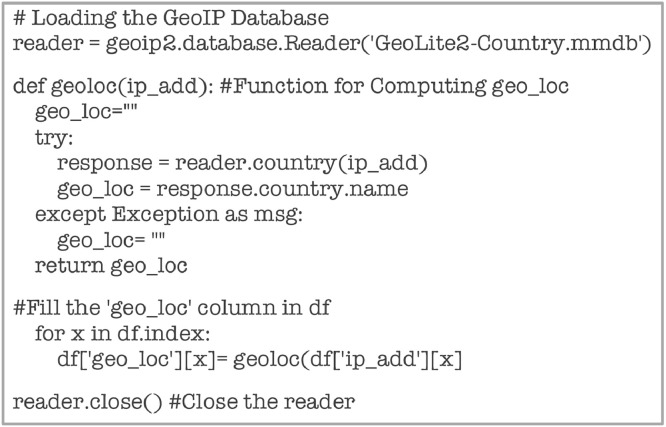
Code snippet for computing ‘geo_loc.

The ‘geo_loc’ attribute, which gives out the country to which the IP Address belongs, is computed from GeoIP Database [6], as given by the code in Fig. 20.

**Fig. 21:**
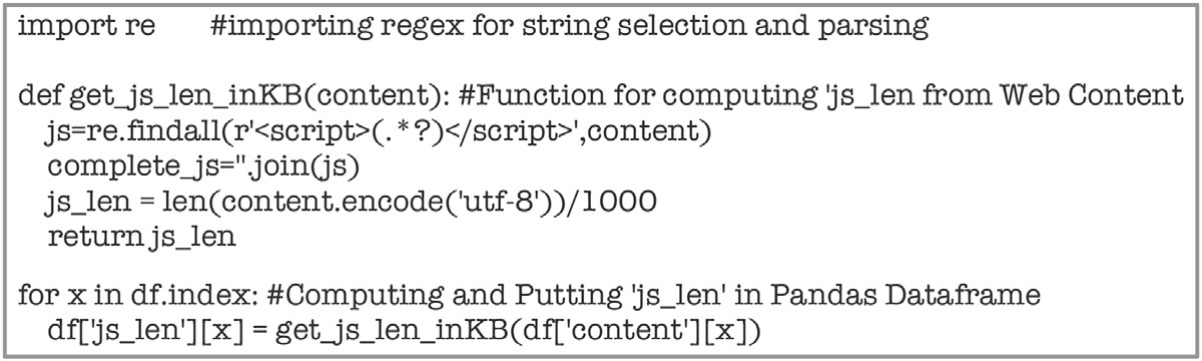
Code snippet for computing ‘js_len.

Attribute ‘js_len’ is computed using the code given in Fig. 21. The JavaScript code, enclosed within ‘<script>*****</script>’ tags are identified and extracted using regex function.

**Fig. 22:**
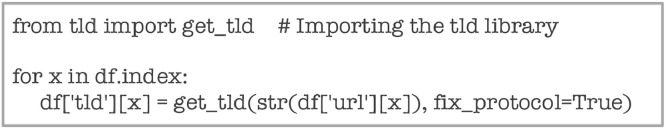
Code snippet for extracting ‘tld’.

Attribute ‘js_obf_len’ requires decoding of the obfuscated JavaScript code before computation. This decoding of obfuscated code is carried out using ‘JavaScript Auto De-Obfuscator’ (JSADO) [7] and Selenium Python library [8]. Code for de-obfuscation is available at [9]. Attribute ‘tld’ is computed from URL using the Python ‘Tld’ library [10]. Code snippet for this extraction is given below in Fig. 22.

**Fig. 23:**
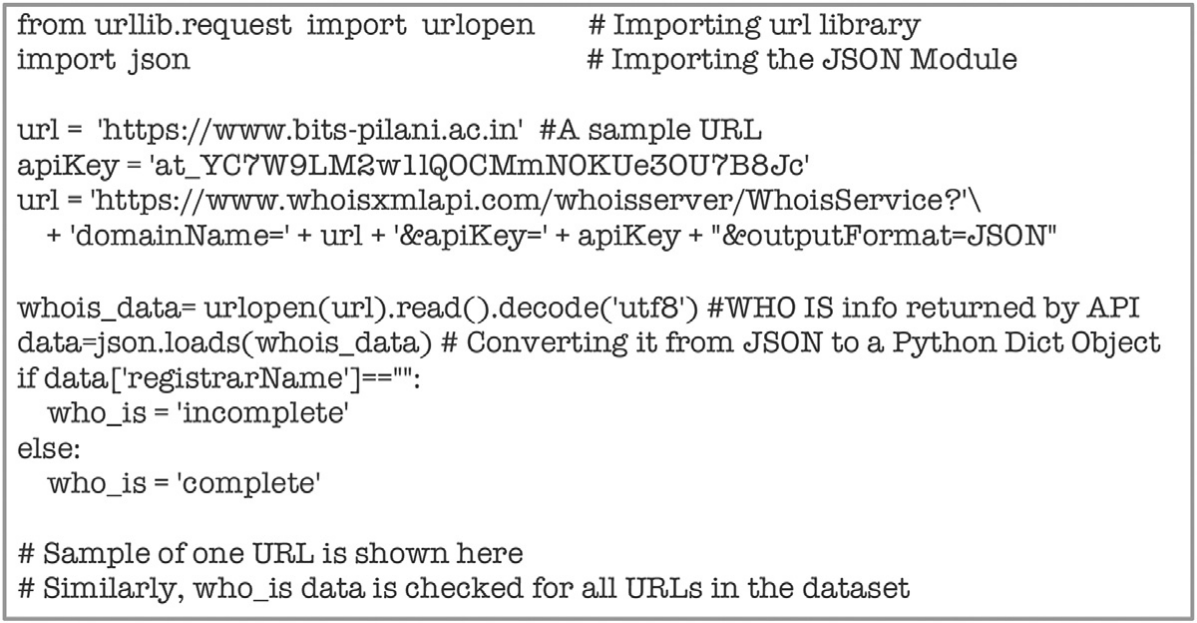
Code snippet for computing ‘who_is.

Attribute ‘who_is’ is computed with the WHOIS API [11] using the code snippet shown below in Fig. 23.

**Fig. 24:**
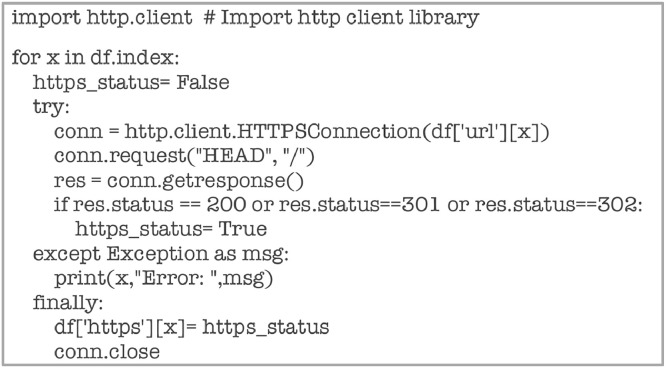
Code snippet for computing ‘https’.

Attribute ‘https’ is computed using the code shown in Fig. 24 below.

**Fig. 25:**
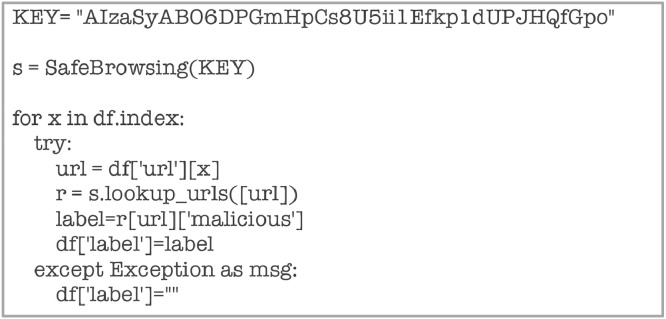
Code snippet for computing Class Labels.

Class labels for this dataset have been generated using the Google Safe Browsing API (refer the sample code for generating labels below in Fig. 25).

The code used for generating and pre-processing this dataset has been hosted online on the Mendeley repository [3], and Kaggle [12] to facilitate future research.

## Ethics Statement

The work did not involve any human subject or animal experiments.

## Declaration of Competing Interest

The author declares that he has no known competing financial interests or personal relationships which have, or could be perceived to have, influenced the work reported in this article.

## Appendix Supplementary Materials

Image, application 1

## Footnotes

2 A webpage is malicious if it has a malware (Cross Site Scripting (XSS), Code injection or Drive by Download based malware), or exhibits behavior like phishing.

3 Profanity Score is a mathematical value given to a group of words based on their goodness/badness. A higher value indicates that a greater number of bad/vulgar words were present.

4 Correlation score gives the relationship between two attributes, with higher score depicting closer relationship.
